# Reinforcing Reinforcement

**DOI:** 10.1371/journal.pbio.1000340

**Published:** 2010-03-23

**Authors:** William Mair

**Affiliations:** Freelance Science Writer, La Jolla, California, United States of America

**Figure pbio-1000340-g001:**
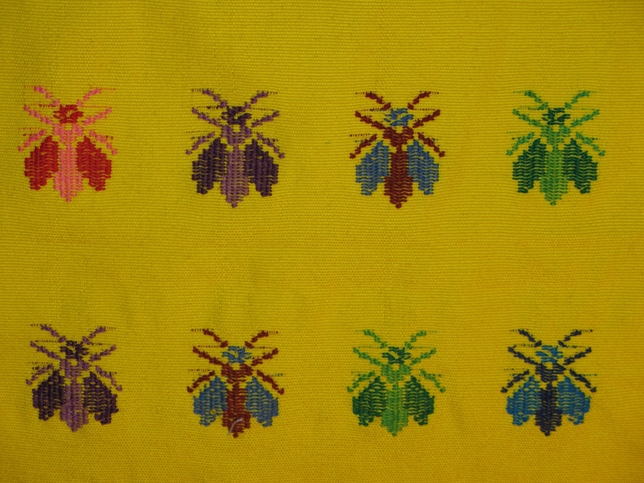
Traditional Guatemalan weaving showing a group of fruit flies. Two species of fruit flies are used to demonstrate that natural selection can influence reproductive isolating mechanisms that occur after mating but before fertilization. Image: Martha Sprigge.

[Fig pbio-1000340-g001]If two populations become geographically isolated from one another, differences in their environments can drive the evolution of new traits over time that eventually result in the emergence of two species where once there was one. Trouble arises however, when individuals from two such populations meet up again before the speciation process is complete. Although partially diverged species can still interbreed, their offspring are often inviable or sterile. The theory of reinforcement suggests that the fitness costs associated with hybrid incompatibility lead to mechanisms, such as mate discrimination, that prevent breeding among partially diverged species. Despite the logical appeal of this explanation, reinforcement remains controversial and empirical studies to support it have produced mixed results.

In this issue of *PLoS Biology*, Daniel Matute describes a new mechanism by which species barriers can be “reinforced.” He focused on two fruit fly species that had diverged on the volcanic slopes of São Tomé, an island off the coast of Africa. Although previous research had failed to identify reinforcement in these species, researchers hadn't looked for mechanisms that prevent the production of less fit hybrids once mating has occurred. The new data demonstrate how reinforcement can occur in the window between mating and fertilization in animals and that such a strategy can rapidly evolve within just a few generations in the laboratory. These results have broad implications for our understanding of reinforcement and how it drives the evolution of new species. They also suggest why scientists have had a hard time proving it occurs in the wild.

Reinforcement relates specifically to changes that occur only in places where species' territories overlap. Demonstrating it in nature therefore requires finding two recently diverged species that co-exist in the same location. Importantly, however, animals interbreeding in this hybrid zone need to be compared to those bred from control groups that still reside in geographical isolation from each other (such isolated populations are termed “allopatric”). Mechanisms that decrease hybridization qualify as reinforcement only if they are seen more within the hybrid zone than outside it (e.g., individuals from within the hybrid zone are choosier about their mates than those from allopatric populations). The sister fruit fly species *Drosophila yakuba* and *Drosophila santomea* display many characteristics that suggest reinforcement has taken place, but until now no evidence for it had been found. *D. yakuba* exists throughout sub-Saharan Africa, while *D. santomea* resides only on São Tomé. It's clear these species recently diverged because they can still generate hybrid progeny, although all males produced by cross-species pairings are sterile. Previous work showed that *D. yakuba* and *D. santomea* have indeed evolved behavioral differences to reduce cross-mating, consistent with reinforcement theory. However, because individuals from allopatric populations exhibited the same behavioral differences as those in the hybrid zone, they were not considered reinforcement.

Matute reasoned that reinforcement might indeed be occurring, but that researchers simply hadn't looked in the right place. He suspected that post-mating mechanisms might be operating to reduce fertilization of eggs from cross-species matings. To test this possibility, he crossed *D. santomea* males to *D. yakuba* females taken either from the hybrid zone or from allopatric populations. Both sets of *D. yakuba* females mated with *D. santomea* males to the same degree, yet females from the hybrid zone produced significantly fewer progeny than those that had never encountered *D. santomea* in the wild. Therefore, reinforcement mechanisms do indeed exist between these species, but instead of reducing how often they mate together they act after the fact to reduce fertilization.

Matute then investigated how this post-mating reinforcement was happening. Rather than fertilizing all available eggs when they mate, female flies store males' sperm and use it gradually to fertilize eggs over several days. Fascinatingly, although the amount of sperm transferred during mating was the same, *D. yakuba* females from the hybrid zone got rid of viable *D. santomea* sperm faster than those that lived in isolation. Females specifically within the hybrid zone have therefore evolved the ability to reject sperm from *D. santomea* in order to reinforce speciation post-mating.

Rejecting inter-species sperm increases the evolutionary success of *D. yakuba* females in two ways. Proteins in *Drosophila* seminal fluid induce behavioral changes in the female that not only stimulate egg production, but also make her less receptive to the advances of other males. Rejecting *D. santomea* sperm quickly therefore benefits females in the hybrid zone not only by reducing the number of sterile male progeny they produce but also by enabling them to mate again sooner. Matute confirmed this by mating *D. yakuba* females to *D. santomea* males before exposing them to a second male from their own species. Females from the hybrid zone were indeed more receptive to the second male than those from allopatric populations, in further support of post-mating reinforcement between these species.

Matute then showed that post-mating reinforcement could evolve rapidly under laboratory conditions. To do this, he housed *D. santomea* males with wild caught *D. yakuba* females from allopatric populations that had never encountered *D. santomea*. Initially, these *D. yakuba* showed no signs of post-mating reinforcement, but after just four generations of forced co-habitation, significant reinforcement developed compared with controls housed separately from *D. santomea*.

Taken together, these data support the evolution of reinforcement strategies within hybrid zones to increase fitness and promote speciation. Furthermore, they demonstrate that reinforcement in animals is not limited to behavioral changes, such as mate choice, but can occur after mating as a result of changes to physiology. Since negative data cannot disprove a theory, these data invite re-evaluation of so-called “cryptic” barriers to gene flow between sister species. They also serve as a compelling reminder that finding no sign of reinforcement in species cohabitating in a hybrid zone is not evidence that it doesn't exist—it may just mean you haven't figured out where to look.

**Matute DR (2010) Reinforcement of Gametic Isolation in** ***Drosophila*** **. doi:10.1371/journal.pbio.1000341**

